# Central retinal thickness changes and risk of neovascular glaucoma after intravitreal bevacizumab injection in patients with central retinal vein occlusion

**DOI:** 10.1038/s41598-022-06121-x

**Published:** 2022-02-08

**Authors:** You Hyun Lee, Yu Cheol Kim

**Affiliations:** Department of Ophthalmology, Keimyung University School of Medicine, 1095 Dalgubeol-daero, Dalseo-gu, Daegu, 42601 Republic of Korea

**Keywords:** Diseases, Health care, Medical research, Pathogenesis, Risk factors

## Abstract

This retrospective study evaluated changes in the central retinal thickness (CRT) and the risk factors for neovascular glaucoma (NVG) after intravitreal bevacizumab injection under a pro re nata (PRN) regimen for macular oedema in 57 eyes with central retinal vein occlusion (CRVO). The clinical characteristics at the time of NVG diagnosis were assessed, and baseline and final clinical characteristics and mean CRT values at 1-, 3-, and 6-month follow-up evaluations were recorded. The incidence of NVG was 21.1%, with the neovascular group (12 eyes) showing poor baseline and final visual acuity, a higher incidence of baseline ischaemic-type CRVO and subretinal fluid, a higher mean CRT at the 1-month follow-up, and a higher number of intravitreal bevacizumab injections during the 6-month follow-up. Nine eyes with NVG (75%) showed a mean CRT < 300 μm at the time of diagnosis. An ischaemic CRVO and higher CRT at the 1-month follow-up were related to the development of NVG in the multivariate analysis. Thus, NVG development in CRVO patients treated with intravitreal bevacizumab injections was associated with an ischaemic CRVO and elevated CRT at the 1-month follow-up; PRN bevacizumab regimens based on CRT or control of macular oedema did not completely prevent NVG development.

## Introduction

Central retinal vein occlusion (CRVO) is a common retinal vascular disorder that usually affects elderly patients and results in vision loss due to macular oedema (MO). CRVO is further divided into ischaemic and non-ischaemic types according to the non-perfusion area^1,2^. Neovascular glaucoma (NVG) is the most serious complication of CRVO, and usually occurs in the ischaemic type^3,4^. NVG is caused by neovascularisation of the anterior chamber angle or iris with eventual angle closure^5^. Vascular endothelial growth factor (VEGF) is known to be a key factor associated with MO and neovascularisation^6^. Funk et al.^7^ demonstrated significantly elevated aqueous humour VEGF levels in CRVO patients with MO. Therefore, intravitreal anti-VEGF injection is considered a mainstay therapy for NVG and MO in CRVO^8,9^.

Aqueous and vitreous VEGF levels have been previously reported to be correlated with the severity of MO^10^. Thus, management of MO may be important for preventing the development of NVG. Early studies showed a low incidence of NVG in patients actively treated with intravitreal anti-VEGF injections for MO in CRVO^11–13^. However, recent studies have reported that anti-VEGF therapy merely delays and does not prevent the development of NVG^14,15^. Rong et al.^16^ also demonstrated that the initial visual acuity (VA), relative afferent pupillary defect, and history of systemic hypertension are predictors of NVG in CRVO, while the initial MO presentation and initial anti-VEGF injection were not. Although initial anti-VEGF injections may not decrease the risk of developing NVG in CRVO patients, the MO or the central retinal thickness (CRT) changes that can reflect the amount of VEGF might be useful for predicting the risk of NVG development in these patients, and anti-VEGF treatment on the basis of CRT and good control of MO may prevent the development of NVG.

Thus, we aimed to evaluate the CRT changes and risk for development of NVG in CRVO patients treated with intravitreal bevacizumab (Avastin, Genentech, Inc., South San Francisco, CA, 1.25 mg/0.05 mL) injections (IVBs).

## Results

### Demographic characteristics

A total of 57 eyes (57 patients) were included in this study, of which 12 eyes showed NVG. The mean patient age was 70 ± 10 years in the neovascular group and 64 ± 11 years in the control group; however, the difference was not statistically significant (*p* > 0.05). The affected eye, sex, and incidence of systemic diseases such as hypertension, diabetes mellitus, hyperlipidaemia, and cerebrovascular accident also did not differ significantly between the groups (*p* > 0.05) (Table 1).

**Table 1:** Demographic and clinical characteristics of the neovascular and control groups among patients with central retinal vein occlusion.

Parameters	Neovascular group (n = 12)	Control group (n = 45)	*p* value
Age (years), mean (SD)	70 (10)	64 (11)	0.118
Sex, n (%) male	7 (58.3%)	24 (53.3%)	1.000
Laterality, n (%) right eye	6 (50.0%)	17 (37.8%)	0.517
Hypertension, n (%)	6 (27.3%)	18 (39.1%)	0.421
Diabetes, n (%)	4 (33.3%)	6 (13.3%)	0.717
Hyperlipidaemia, n (%)	1 (8.3%)	5 (11.1%)	1.000
Cerebrovascular accident, n (%)	0 (0.0%)	0 (0.0%)	1.000
Type of CRVO, ischaemic type, n (%)	9 (75.0%)	8 (17.8%)	0.001*
Baseline lens status, phakic, n (%)	8 (66.7%)	36 (80.0%)	0.328
Presence of baseline SRF, n (%)	11 (91.7%)	24 (53.3%)	0.015*
Duration of visual disturbance, days, mean (SD)	9 (5)	11 (6)	0.265
Total number of intravitreal injections during the past 6 months, mean (SD)	3.67 (0.78)	2.43 (1.17)	0.001*
Interval between the doses, month, mean (SD)	2.0 (0.8)	2.1 (1.2)	0.616
Total follow-up period, month, mean (SD)	29 (7)	13 (8)	0.001*
Baseline best-corrected visual acuity, logMAR (SD)	1.01 (0.23)	0.76 (0.50)	0.017*
Baseline intraocular pressure, mmHg, mean (SD)	15.75 (1.82)	14.36 (2.80)	0.108
Baseline central retinal thickness, μm, mean (SD)	520.42 (65.40)	465.33 (181.12)	0.101
Final best-corrected visual acuity, logMAR (SD)	1.43 (0.36)	0.60 (0.33)	0.000*
Final intraocular pressure, mmHg, mean (SD)	17.08 (3.85)	14.84 (3.78)	0.075
Final central retinal thickness, μm, mean (SD)	343.67 (146.74)	328.84 (162.49)	0.776

*CRVO* central retinal vein occlusion, *SRF* subretinal fluid.

*Statistically significant by independent two-sample t-test or Pearson’s chi-square test.

### Baseline and final clinical characteristics in the neovascular and control groups

Ischaemic-type CRVO was more frequently observed in the neovascular group (75.0%) than in the control group (17.8%, *p* = 0.001). Subretinal fluid (SRF) was more often identified in the neovascular group than in the control group (91.7% vs. 53.3%, *p* = 0.015). The shorter duration of visual disturbance was observed in the neovascular group (9 ± 5 vs. 11 ± 6 days); however, it was statistically insignificant (*p* > 0.05). The logMAR best-corrected visual acuity (BCVA) was significantly worse in the neovascular group (1.01 ± 0.23) than in the control group (0.76 ± 0.50, *p* = 0.017). The total follow-up period was significantly longer in the neovascular group (29 ± 7 months vs. 13 ± 8 months, *p* = 0.001). The total number of IVBs during the 6-month period was significantly higher in the neovascular group (3.67 ± 0.78 vs. 2.43 ± 1.17, *p* = 0.001). The interval between the doses showed an insignificant difference between the two groups (*p* > 0.05). The mean CRT was higher in the neovascular group (520.42 ± 65.40 μm) than in the control group (465.33 ± 181.12 μm); however, the difference was not statistically significant (*p* > 0.05). Furthermore, the lens status and mean intraocular pressure (IOP) were not significantly different between the two groups (*p* > 0.05). The final logMAR BCVA was 1.43 ± 0.36 in the neovascular group and 0.60 ± 0.33 in the control group, and the difference was statistically significant. The differences in the final mean IOP and CRT were not significant (*p* > 0.05) (Table 1).

### Central retinal thickness changes

**Figure 1:**
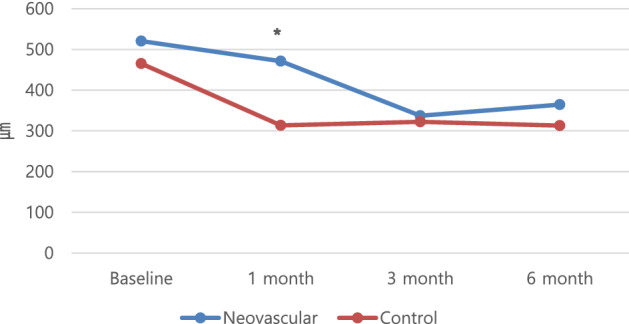
Changes in central retinal thickness after intravitreal bevacizumab injections in the neovascular and control groups of patients with central retinal vein occlusion. *Statistically significant by independent two-sample t-tests.

Both the neovascular and control groups showed a reduced CRT after the initial visit. The two groups showed statistically insignificant differences in the 3- and 6-month follow-up examinations (*p* > 0.05), but the CRT at the 1-month follow-up examination was significantly higher in the neovascular group (471.21 ± 82.37 vs. 313.60 ± 133.49 μm, *p* = 0.001) (Fig. 1).

### Clinical characteristics at the time of neovascular glaucoma diagnosis and the clinical prognosis of neovascular glaucoma patients

The logMAR BCVA was 1.53 ± 0.47. The mean IOP was 39.25 ± 12.59 mmHg. The mean CRT was 281.08 ± 57.69 μm, and the CRT values were ≥ 300 μm in three patients (25.0%). The mean interval between the onset of NVG and the initial visit was 16 ± 6 months. The mean onset of NVG after the last intravitreal injection was 7 ± 2 months. Among these 12 NVG patients, 5 patients (41.7%) subsequently underwent Ahmed valve implantation surgery to control the IOP. (Table 2).

**Table 2:** Clinical characteristics of neovascular glaucoma patients at the time of diagnosis and clinical prognosis of neovascular glaucoma patients.

Parameters	Neovascular group (n = 12)
Best-corrected visual acuity, logMAR (SD)	1.53 (0.47)
Intraocular pressure, mmHg, mean (SD)	39.25 (12.59)
Central retinal thickness, μm, mean (SD)	281.08 (57.69)
≥ 300 μm, n (%)	3 (25)
Onset of neovascular glaucoma after the CRVO, months, mean (SD)	16 (6)
Onset of neovascular glaucoma after the last injection, months, mean (SD)	7 (2)
Number of patients received Ahmed valve implantation surgery, n (%)	7 (2)

*CRVO* central retinal vein occlusion.

### Analysis of the factors associated with development of neovascular glaucoma

In the binary logistic regression analysis, ischaemic-type CRVO (*p* = 0.006) and CRT at the 1-month follow-up (*p* = 0.007) were significantly associated with the development of NVG in CRVO patients treated with IVBs (Table 3).

**Table 3:** Multivariate analysis of possible factors associated with neovascular glaucoma in central retinal vein occlusion patients.

Parameters	Adjusted OR	95% CI	*p* value
Age			0.151
Baseline best-corrected visual acuity			0.695
Baseline intraocular pressure			0.194
Baseline central retinal thickness			0.853
Baseline presence of subretinal fluid			0.145
Ischaemic type	13.087	2.120–80.781	0.006*
Number of intravitreal bevacizumab injections during the past 6 months			0.392
1-month follow-up central retinal thickness	1.008	1.003–1.014	0.007*

*OR* odds ratio, CI confidence interval.

*Statistically significant by binary logistic regression analysis with backward elimination method (R^2^ = 0.560, *p* = 0.831 by the Hosmer–Lemeshow test for goodness of fit).

## Discussion

NVG is a major complication of CRVO and may lead to irreversible visual impairment. IVB is widely used for MO treatment in CRVO^17^. This therapy is also used to induce regression of anterior segment neovascularisation in NVG^18^. The NVG incidence rate was 21.1% (12 eyes of 57 eyes) in this study. It was higher than the previous studies (6.8 and 13%)^15,16^. This discrepancy is because the three patients of initially non-ischaemic type CRVO developed NVG in the present study. Hayreh et al.^19^ demonstrated about the non-ischaemic type converted into ischaemic type CRVO during the follow-up. The consecutive fluorescein angiography was not performed in this study; however, this event could have happened and resulted in a higher incidence of development of NVG. The incidence of NVG shown in the present study also supports the findings of previous studies stating that intravitreal anti-VEGF injection could not prevent the development of NVG. This is because the pro re nata (PRN) regimen used in this study tended to show an inadequate therapeutic effect in comparison with fixed monthly injections, which resulted in higher VEGF levels and consequent NVG development. This is consistent with the findings of the RAVE trial, which showed NVG development during the PRN regimen after the completion of nine monthly injections^14^.

The current study retrospectively analysed the baseline and final clinical characteristics of CRVO patients treated with IVB who were divided into a neovascular group and a control group. At baseline, the neovascular group showed a worse logMAR BCVA. This is because SRF and ischaemic CRVO were more frequently observed in the neovascular group. Celik et al.^20^ reported that SRF in CRVO occurs as a result of external limiting membrane breakdown, in which the movement of intraretinal fluid into the subretina and the SRF itself is related to poor VA prognosis. The severe structural damage in ischaemic CRVO might be related to a higher incidence of SRF and worse logMAR BCVA. The final logMAR BCVA was also poor in the neovascular group.

In the present study, the mean interval between the last injection and NVG onset was 7 months, and this result is consistent with that reported by Rong et al.^16^. The presence of an ischaemic-type CRVO and a higher CRT at the 1-month follow-up examination were risk factors for NVG development. BCVA is known to be the most accurate predictor of NVG development in CRVO^3^. A significantly poorer BCVA was noted in the neovascular group; however, it was not a risk factor in this study. This is because the current study only enrolled patients with CRVO who had MO and underwent IVB treatment at the initial visit. An ischaemic CRVO can be easily considered to be a risk factor for the development of NVG, since Hayreh et al.^21^ reported a higher cumulative incidence rate of NVG in cases with an ischaemic CRVO (40%) compared to cases with a non-ischaemic CRVO (10%). However, the correlation between a higher CRT at 1 month and the development of NVG is somewhat interesting. The baseline CRT was higher in the neovascular group; however, it was statistically insignificant and was not a risk factor for NVG development. On the basis of these findings, the CRT at the 1-month follow-up could represent a poor response to the initial IVB, indicating that the decrease in VEGF levels with just a single injection was not sufficient and that this limited effectiveness could be related to the future development of NVG. The CRTs at the 3- and 6-month follow-up examinations were not significantly different between the two groups, which could be attributed to the fact that the neovascular group had significantly more intravitreal injections during the follow-up period.

The current study also evaluated the clinical characteristics at the time of NVG diagnosis. Interestingly, the mean CRT was 281.08 ± 57.69 μm. Moreover, only three patients showed CRT ≥ 300 μm. This finding was in contrast to our expectations since NVG is related to high levels of VEGF, and a higher CRT should be observed at the time of NVG diagnosis. Several reports have mentioned that vitreous VEGF levels correlate with CRT in diabetic macular oedema (DMO) and VEGF diffusion from the posterior toward the anterior segment^6,22,23^. However, a contradiction between MO and neovascularisation has also been observed in the relationship between DMO and diabetic retinopathy (DR). In the VIBIM study^24^, which used a treat-and-extend regimen trial with aflibercept for treating DMO, DR was aggravated in some cases with a reduced number of anti-VEGF injections, although the DMO was successfully controlled, suggesting that anti-VEGF cannot suppress the aggravation of DR for more than 4 months, consistent with Protocol S at DRCR.net^25^. These results imply that neovascularisation does not always accompany MO, although both are affected dominantly by the same cytokine (VEGF), and that anti-VEGF treatment targeting MO control does not satisfactorily suppress neovascularisation, regardless of the underlying retinal diseases.

Tripathi et al.^26^ demonstrated that the ciliary epithelium produces VEGF and plays an important role in the development of NVG by increasing aqueous humour VEGF levels. The rate of increase in VEGF levels in the aqueous humour and vitreous humour could be different after cessation of IVB. We hypothesise that the early poor response to initial IVB could have induced faster production in the ciliary epithelium than in the vitreous after the cessation of IVB. Further studies are needed to measure the actual concentration of VEGF levels in the aqueous humour and vitreous humour after IVB cessation.

The current study had some limitations. First, the retrospective design may have resulted in a patient selection bias. Second, this study included a relatively small number of patients with NVG. Third, the comparison of CRT for six months could be considered a short period of time, and a prospective approach with a longer period is needed to validate the findings. Fourth, the mean follow-up period of the control group was shorter than the mean interval period of NVG development. The NVG patients might have been overlooked in the control group. Finally, the anterior chamber angle was not assessed in this study. Thus, NVG patients who only had neovascularisation over the angle might have been overlooked. However, we believe that this study will provide valuable information about NVG risk factors in CRVO patients treated with IVB.

In conclusion, the incidence of ischaemic-type CRVO and CRT at the 1-month follow-up was related to the development of NVG in CRVO patients treated with IVBs, and a PRN regimen of anti-VEGF agents on the basis of CRT or good control of MO cannot prevent the development of NVG.

## Methods

### Study design and subjects

This study was designed as a retrospective, comparative case series conducted at a single hospital. The study adhered to the tenets of the Declaration of Helsinki and was approved by the Institutional Review Board of Keimyung University Dongsan Hospital (IRB no. 2021-08-061), and the need for informed consent was waived due to the retrospective nature of the study. We retrospectively reviewed the electronic medical records of treatment-naïve CRVO patients treated with IVBs who were followed up for at least six months from January 2018 to December 2020. The subsequent IVBs were performed in accordance with a PRN regimen that met at least one of the following criteria; (1) presence of MO (CRT > 300 µm), (2) presence of foveal cyst lesions with decreased BCVA and distortion of foveal contour. The exclusion criteria were as follows: a previous history of intravitreal anti-VEGF or steroid treatment; change of treatment options (intravitreal triamcinolone injection or intravitreal dexamethasone implants) during the follow-ups; history of pars plana vitrectomy or cataract surgery within 6 months preceding the start of the trial; or the presence of other retinal diseases that could cause decreased BCVA, such as epiretinal membrane, a grade exceeding severe non-proliferative DR, rhegmatogenous retinal detachment, age-related macular degeneration, or uveitis. NVG was diagnosed when the clinician noted neovascularisation of the iris with high IOP by Goldman tonometry. The patients were divided into neovascular and control groups. Panretinal photocoagulation was performed in all patients with NVG at the time of diagnosis. The clinical characteristics at the time of NVG diagnosis and the risk factors for NVG were also assessed.

### Clinical data collection

At the initial visit, demographic characteristics, including age, sex, laterality, presence of hypertension, diabetes, cerebrovascular attack, and hyperlipidaemia were assessed. The duration of visual disturbance was also assessed in each patient. Baseline lens status, baseline and final BCVA (logMAR), and IOP were recorded. The ischaemic type was defined as > 10 disc areas of retinal capillary non-perfusion detected using fluorescein angiography (HRA-2; Heidelberg Engineering, Heidelberg, Germany). The presence of SRF and CRT was measured using optical coherence tomography (DRI OCT-1; Topcon, Tokyo, Japan). The CRTs at the 1-, 3-, and 6-month follow-up assessments and the total number of IVBs over the 6-month follow-up period were also assessed. The interval between the doses was also recorded. To evaluate the clinical characteristics at the time of NVG diagnosis, the following parameters were evaluated: BCVA, IOP measured by Goldman tonometry, CRT, interval between the baseline and the development of NVG, and interval between the last IVB and the diagnosis of NVG.

### Statistical methods

Data were calculated as means ± standard deviation (SD) or n (%; e.g. the number of eyes). Statistical analyses were performed using the Statistical Package for the Social Sciences (SPSS) version 12.0 (IBM, Chicago, IL, USA). The between-group differences in age; baseline and final BCVA; baseline and final IOP; baseline, 1-month, 2-month, 6-month, and final CRT; total follow-up period; and total number of IVBs during the 6-month period were compared using independent t-tests. Categorical variables such as sex, affected eye, baseline lens status, presence of systemic diseases, type of CRVO (ischaemic vs. non-ischaemic), and presence of baseline SRF were compared using Pearson’s chi-square tests. Binary logistic regression analysis was used to identify the factors associated with NVG development.

## Data Availability

The datasets generated and/or analysed during the present study are available from the corresponding author upon reasonable request.
